# Non-invasive measurement of choroid plexus apparent blood flow with arterial spin labeling

**DOI:** 10.1186/s12987-020-00218-z

**Published:** 2020-09-22

**Authors:** Li Zhao, Manuel Taso, Weiying Dai, Daniel Z. Press, David C. Alsop

**Affiliations:** 1 Key Laboratory for Biomedical Engineering of Ministry of Education, College of Biomedical Engineering & Instrument Science, Zhejiang University, Hangzhou, Zhejiang China; 2 Radiology, Beth Israel Deaconess Medical Center and Harvard Medical School, Boston, MA USA; 3 Computer Science, State University of New York At Binghamton, Binghamton, NY USA; 4 Neurology, Beth Israel Deaconess Medical Center and Harvard Medical School, Boston, MA USA

**Keywords:** Choroid plexus, Cerebrospinal fluid, Perfusion, Magnetic resonance imaging, Arterial spin labeling

## Abstract

**Background:**

The choroid plexus is a major contributor to the generation of cerebrospinal fluid (CSF) and the maintenance of its electrolyte and metabolite balance. Here, we sought to characterize the blood flow dynamics of the choroid plexus using arterial spin labeling (ASL) MRI to establish ASL as a non-invasive tool for choroid plexus function and disease studies.

**Methods:**

Seven healthy volunteers were imaged on a 3T MR scanner. ASL images were acquired with 12 labeling durations and post labeling delays. Regions of the choroid plexus were manually segmented on high-resolution T_1_ weighted images. Choroid plexus perfusion was characterized with a dynamic ASL perfusion model. Cerebral gray matter perfusion was also quantified for comparison.

**Results:**

Kinetics of the ASL signal were clearly different in the choroid plexus than in gray matter. The choroid plexus has a significantly longer T_1_ than the gray matter (2.33 ± 0.30 s vs. 1.85 ± 0.10 s, p < 0.02). The arterial transit time was 1.24 ± 0.20 s at the choroid plexus. The apparent blood flow to the choroid plexus was measured to be 39.5 ± 10.1 ml/100 g/min and 0.80 ± 0.31 ml/min integrated over the posterior lateral ventricles in both hemispheres. Correction with the choroid plexus weight yielded a blood flow of 80 ml/100 g/min.

**Conclusions:**

Our findings suggest that ASL can provide a clinically feasible option to quantify the dynamic characteristics of choroid plexus blood flow. It also provides useful reference values of the choroid plexus perfusion. The long T_1_ of the choroid plexus may suggest the transport of water from arterial blood to the CSF, potentially providing a method to quantify CSF generation.

## Background

Cerebrospinal fluid (CSF) plays essential roles in development, physiology, and pathology of the brain. Beyond the protection of the brain from trauma, CSF transports, and regulates molecules that are essential for neuronal metabolism [1]. On the one hand, CSF and blood-CSF barrier control the entry of iron, metabolites, electrolytes, and proteins into the brain [2]. On the other hand, CSF also serves as a drainage pathway of metabolic waste [3, 4]. The CSF flowing through the perivascular spaces may remove amyloid-beta and other toxic molecules by actively exchanging with the interstitial fluid [5–7]. Recent studies further suggested that dysfunction of brain clearance mechanisms could be responsible for the accumulation of amyloid-beta and tau protein in Alzheimer’s Disease (AD) [8–11].

CSF is mainly secreted by the choroid plexus, most of which is attached to the walls of the lateral ventricles. The anterior choroidal artery originating from the internal cerebral artery and the posterior choroidal artery originating from the posterior cerebral artery provide blood supply to the choroid plexus in the lateral ventricle. Distinct from the blood–brain barrier, capillaries of the choroid plexus are fenestrated and water exchanges freely between the choroid plexus stroma and the blood. The boundary between the choroid plexus and the CSF is composed of a monolayer of choroidal epithelial cells that have tight junctions between each other and form the blood-CSF barrier. Because of this unique vascular structure, the perfusion of the choroid plexus may show special characteristics compared to other regions of the brain. In addition, early studies showed that the choroid plexus blood flow was associated with CSF production [12, 13]. Therefore, the characteristics of choroid plexus perfusion would be of great interest.

However, studies of the choroid plexus perfusion were rarely performed, which were partly obstructed by the shortage of safe methods. A number of early studies were performed with radioactive tracers and animal models. Townsend et al. [14], Faraci et al. [15–17], Williams et al. [18, 19], Szmydynger-Chodobska [20], and Ennis et al. [21] injected radioactive microspheres and measured radiation doses of the choroid plexus on sacrificed animals. After years of medical imaging developments, only a few studies reported choroid plexus perfusion or permeability in human subjects using contrast-enhanced MRI with Gadolinium-based contrast agents (GBCA) [22, 23]. However, these studies didn’t provide absolute blood flow, which is challenging with GBCA because of the nonlinear dependence of signal on concentration and tissue distribution. More rapid outflow in the highly vascular choroid plexus may limit its accuracy. Concerns about the deposition of Gadolinium in tissue may also limit the use of the technique, especially in research studies with little individual benefit to subjects [24, 25]. Therefore, it would be highly desirable to develop a clinically feasible and totally non-invasive method for the measurement of choroid plexus perfusion.

Arterial spin labeling (ASL) perfusion MRI is an appealing non-invasive and quantitative option for the measurement of choroid plexus apparent blood flow. In an ASL scan, the endogenous arterial blood water is labeled by radiofrequency pulses. When the labeled blood flows into the brain tissue, it results in reduced signal compared to a control scan with non-labeled blood. Through the measurement of the signal change between the control and label scans, ASL is able to quantify the blood flow using the endogenous arterial blood water signal. Therefore, ASL provides a safe technique without contrast agent administration and a clinically feasible option for characterizing choroid plexus perfusion.

Clearly visible choroid plexus signal has been observable in brain ASL imaging studies for many years, such as the Fig. 10 of Dai et al. [26], the Fig. 1 of Mutsaerts et al. [27], and the Fig. 9 of Amukotuwa et al. [28]. The ASL signal of the choroid plexus has also been observed in patients with Alzheimer’s Disease [29]. In a recent resting-state functional study with ASL, our results further indicated that the ASL signal of the choroid plexus didn’t participate in flow fluctuations present in brain networks [30]. However, sources of high ASL signal in the choroid plexus were not discussed in the above work.

Recently, we reported the feasibility of detecting choroid plexus perfusion using ASL in abstract form [31, 32], but the blood flow quantification methods were not fully developed. Johnson et al. [33] implemented this method to evaluate perfusion changes after angiogenesis, but they used a single delay ASL experiment, which couldn’t provide the dynamic characteristics of choroid plexus perfusion. Evans et al. [34] reported a successful detection of CSF generation on animal models using a pulsed ASL with a long echo time. However, the human studies in their work showed unexpected signal magnitudes and kinetics.

In this work, we sought to establish ASL as a tool to quantify the dynamic characteristics of choroid plexus perfusion. The perfusion characterization can serve as a preliminary reference for studies of physiological and pathological modulation of choroid plexus function.

## Methods

### Subject recruitment

Seven healthy adult volunteers (5 females, mean age 35 ± 18 years) were recruited for this study. All subjects were imaged following a protocol approved by the institutional committee on clinical investigations. Written informed consent was obtained from all subjects prior to MRI scanning.

### MR imaging protocol

MR imaging was performed on a GE 3 Tesla Discovery MR750 scanner (GE Healthcare, Chicago, IL) using a body coil for transmission and a 32-channel head array for signal reception. High-resolution T_1_ weighted images and 12 different ASL images were acquired in each subject.

First, whole brain T_1_-weighted images were acquired to segment the choroid plexus. A 3D inversion-prepared spoiled gradient echo sequence was used to acquire high-resolution T_1_-weighted images. Scan parameters included: inversion time (TI) of 450 ms, echo time (TE) of 3.24 ms, and repetition time (TR) of 8.24 ms. Parallel imaging was used to reduce the scan time to approximately 4 min 20seconds. The spatial resolution was 0.94 mm × 0.94 mm × 1 mm.

Next, 12 ASL images were acquired to quantify the dynamic characteristics of the choroid plexus perfusion. The label/control of each ASL acquisition was performed using the unbalanced pseudo-continuous arterial spin labeling (pCASL) technique [26]. Arterial blood was labeled with repeated Hann RF pulses, and static tissue signal was suppressed by background suppression pulses interleaved with ASL label/control pulses [35]. ASL images were acquired with a stack-of-spiral Fast Spin Echo acquisition. Each spiral arm used a readout of 8 ms with bandwidth 62.5 kHz, and three interleaved arms were used. For a field-of-view 220 mm, this setting resulted in an in-plane resolution of 4 mm × 4 mm. 32 slices were acquired with slice thickness 4 mm. In each ASL scan, four pairs of control and label scans were acquired to improve the signal-to-noise ratio. TE was 14.7 ms, and TR was from 4.24 to 9.19 s. At the end of each ASL scan, one proton density reference image was acquired with a saturation time of 2 s. The total time of ASL scans was about 40 min.

The above ASL acquisition was performed at 12 different observation times (OT), including blood labeling durations (LD) and post-labeling delays (PLD) listed in Table 1. This design was able to track the inflow and wash-out process of the labeled arterial blood, similar to a previous work [36]. The minimum PLD was chosen to be 0.7 s, which was assumed to be shorter than the arterial transit time [35]. At such short delays, a part of the labeled blood was still in the large arteries when the ASL images were acquired. To quantify the perfusion signals more accurately, a set of vessel suppression pulses [35] was implemented right before the acquisition to remove the labeled blood signals in the large arteries.

**Table 1:** Blood labeling duration (LD) and post-labeling delay (PLD) designs in the experiments

OT	t1	t2	t3	t4	t5	t6	t7	t8	t9	t10	t11	t12
LD (s)	1.5	2.0	2.5	3.0	3.5	4.0	4.0	4.0	4.0	4.0	4.0	4.0
PLD (s)	0.7	0.7	0.7	0.7	0.7	0.7	1.2	1.7	2.2	2.7	3.2	3.7

### MR image processing

ASL images were reconstructed by the manufacturer’s online algorithm. Pair-wise subtraction between label and control data was performed followed by summing of the ASL difference images. ASL images were reconstructed by a 1D Fourier transform along the slice direction, and a gridding operator and 2D Fourier transform in each slice. The proton density reference images were reconstructed in the same manner. At each OT, the ASL and proton density images were scaled with the same factor to maximize the visual dynamic range, when they were exported to DICOM files.

Images were preprocessed using Statistical Parametric Mapping (SPM12) software (Wellcome Trust Centre for Neuroimaging, London) with default settings. The 12 ASL images were realigned for each subject. Since the proton density image was acquired immediately after the ASL image, we assumed negligible movements between the ASL image and the following proton density image. Therefore, the proton density image was aligned using the same parameters obtained in the alignment of the corresponding ASL image.

The 12 realigned ASL images were co-registered to the high-resolution T_1_-weighted images for each subject to avoid potential partial volume effects, because the ASL images have lower resolution than the T_1_-weighted images. T_1_-weighted images were segmented into the gray matter, the white matter, the CSF, the skull, and the fat regions using the segmentation tool from SPM12. The mean of the 12 realigned ASL images was registered with the gray matter probability map. The individual ASL and proton density images were then transformed to the T_1_-weighted image space using the above generated transformation matrix.

**Fig. 1:**
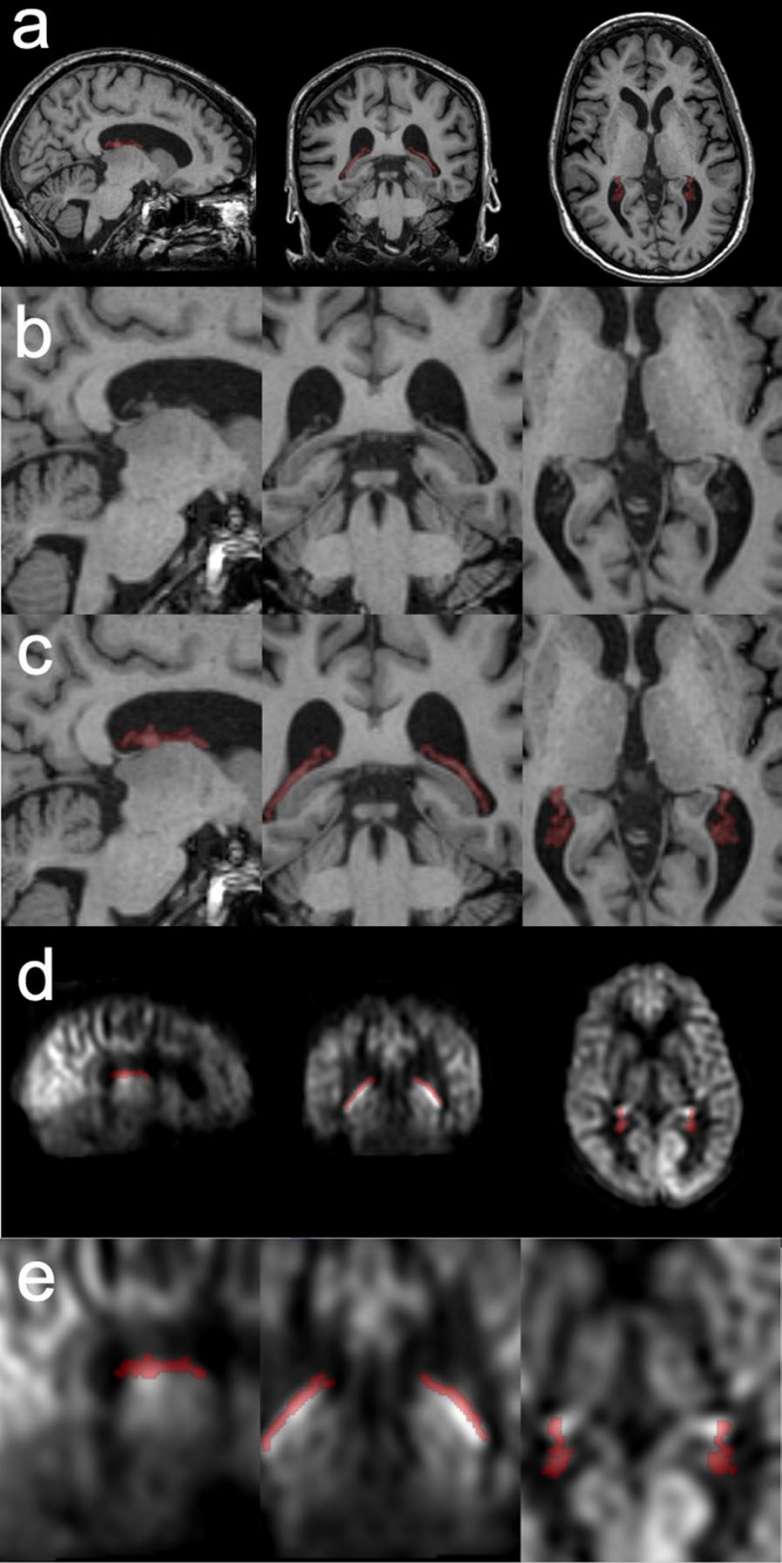
Choroid plexus regions segmented based on T_1_-weighted images. The choroid plexus region was segmented manually in the lateral ventricle on the high-resolution T_1_ weighted images (**a**–**c**). The ASL images were registered to the gray matter of the T_1_-weighted images (**d**, **e**)

The choroid plexus regions were manually segmented based on the T_1_-weighted images using ITK-SNAP [37]. The most readily recognized choroid plexus regions were selected in the posterior part of the lateral ventricles, Fig. 1. The manual segmentation was performed on axial, coronal, and sagittal planes and was checked in a 3D view by a scientist (L.Z.) with 10 years MR neuroimaging experience. The gray matter regions were selected by a threshold of 0.5 on the gray matter probability map. The image overlay was rendered using fsleyes (Wellcome Centre for Integrative Neuroimaging (FMRIB), University of Oxford, UK) without interpolation.

### Perfusion characteristics quantification

The blood flow, arterial transit time, and T_1_ of the gray matter and the choroid plexus regions was quantified using a standard dynamic ASL model [35].

$$\frac{{{\Delta }M}}{{M_{0} }} = 2\alpha fT_{1} e^{{ - \delta /T_{1a} }} \left( {e^{{ - max\left( {w - \delta ,0} \right)/T_{1} }} - e^{{ - max\left( {w + \tau - \delta ,0} \right)/T_{1} }} } \right)$$

where ${\Delta }M$ is the ASL signal. Pixel-wise ASL signal was used to obtain the spatial distribution of perfusion. The mean ASL signal of the choroid plexus or the gray matter regions was used to calculate average perfusion. $\alpha = \alpha_{1} \alpha_{2}$ is the labeling efficiency; $\alpha_{1} = 0.8$ is the inversion efficiency ratio of the arterial blood [26]; $\alpha_{2} = 0.75$ is the background suppression efficiency [38]. $T_{1a} = 1.65\,{\text{s}}$ is the longitudinal relaxation rate of the arterial blood [39]. $w$ is the post-labeling delay, and $\tau$ is the labeling duration, Table 1. In this study, a constrained nonlinear least-squares fit (Scipy1.4, optimize.least_squares) was used to quantify three parameters: $f$ is the blood flow, constrained by a lower bound of 10 ml/100 g/min; $\delta$ is the arterial transit time, constrained by a lower bound of 0.7 s [35], and $T_{1}$ is the longitudinal relaxation rate of the labeled water after it is transported from the arterial blood to local tissue, constrained by an empirically selected lower bound of 1 s. The goodness of fit was measured by a coefficient of determination R^2^.

$M_{0}$ is the equilibrium magnetization of arterial blood calculated from the proton density image.

$$M_{0} = \frac{{M_{PD} }}{{\lambda \left( {1 - e^{{ - T_{sat} /T_{1g} }} } \right)}}$$

where $M_{PD}$ is the mean signal of the gray matter region and is calculated from the proton density image corresponding to the ASL image at each OT. $\lambda = 0.9$ is the brain blood partition coefficient [35], which is used to approximate the unknown choroid plexus blood partition coefficient. $T_{sat} = 2\,{\text{s}}$ is the saturation recovery time in the proton density image scan, and $T_{1g} = 1.5\,{\text{s}}$ is the longitudinal relaxation rate of the gray matter [35, 40]. The same $M_{0}$ was also used to quantify the perfusion of the gray matter and the choroid plexus, which was equivalent to using the gray matter as reference tissue [41].

Since the choroid plexus and the CSF intermingle at a microscopic level, it is not possible to exclude a partial volume of CSF in the choroid plexus voxels at any achievable MR imaging resolution. The low resolution of ASL introduces severe partial volume effect. Previous work has proposed partial volume corrections for ASL images based on volumetric [42–44] or surface [45] segmentations. However, choroid plexus segmentation has been rarely studied, and the available method showed limited accuracy [46]. Therefore, the blood flow, $f \left( {{\text{ml}}/100\,{\text{g}}/{\min}} \right){ }$, quantified with the ASL model above is an apparent blood flow that includes partial volume effects unavoidably. The apparent blood flow was used because it can be readily compared to ASL measured perfusion in the brain. Assuming no ASL signal contribution from the existing CSF, the total blood flow of choroid plexus volume in the left and right posterior lateral ventricles can be calculated according to

$$F = \mathop \sum \limits_{n} w_{n} v\rho F_{n} = \rho v\mathop \sum \limits_{n} f_{n} = \rho vNf$$

where F is the total blood flow (ml/min), $w_{n}$ represents the partial volume effect in the nth voxel, which is the volume percentile of both the choroid plexus tissue and the potential CSF generated with labeled blood in the voxel. $v$ is the volume of each voxel, and $w_{n} v$ is the choroid plexus volume in the nth voxel. $\rho = 1.08\,{\text{g}}/{\text{ml}}$ is the choroid plexus density, assuming to be the same as the brain parenchyma [47]. $F_{n}$ is the actual blood flow (ml/100 g/min) in the choroid plexus. On the contrary, $f_{n} = w_{n} F_{n}$ is voxel-wise apparent blood flow measured in ASL experiments, including partial volume effects. $f$ is the average apparent blood flow of the choroid plexus, which contains the partial volume effect. $N$ is the number of voxels in the choroid plexus.

### Statistical analysis

The mean values and standard deviations of the apparent blood flow, arterial transit time, and T_1_ were calculated across subjects. Non-parametric and paired comparisons of the above parameters were compared using the Wilcoxon sign-rank method (Scipy1.4, stats.wilcoxon) between the gray matter and the choroid plexus regions.

## Results

**Fig. 2:**
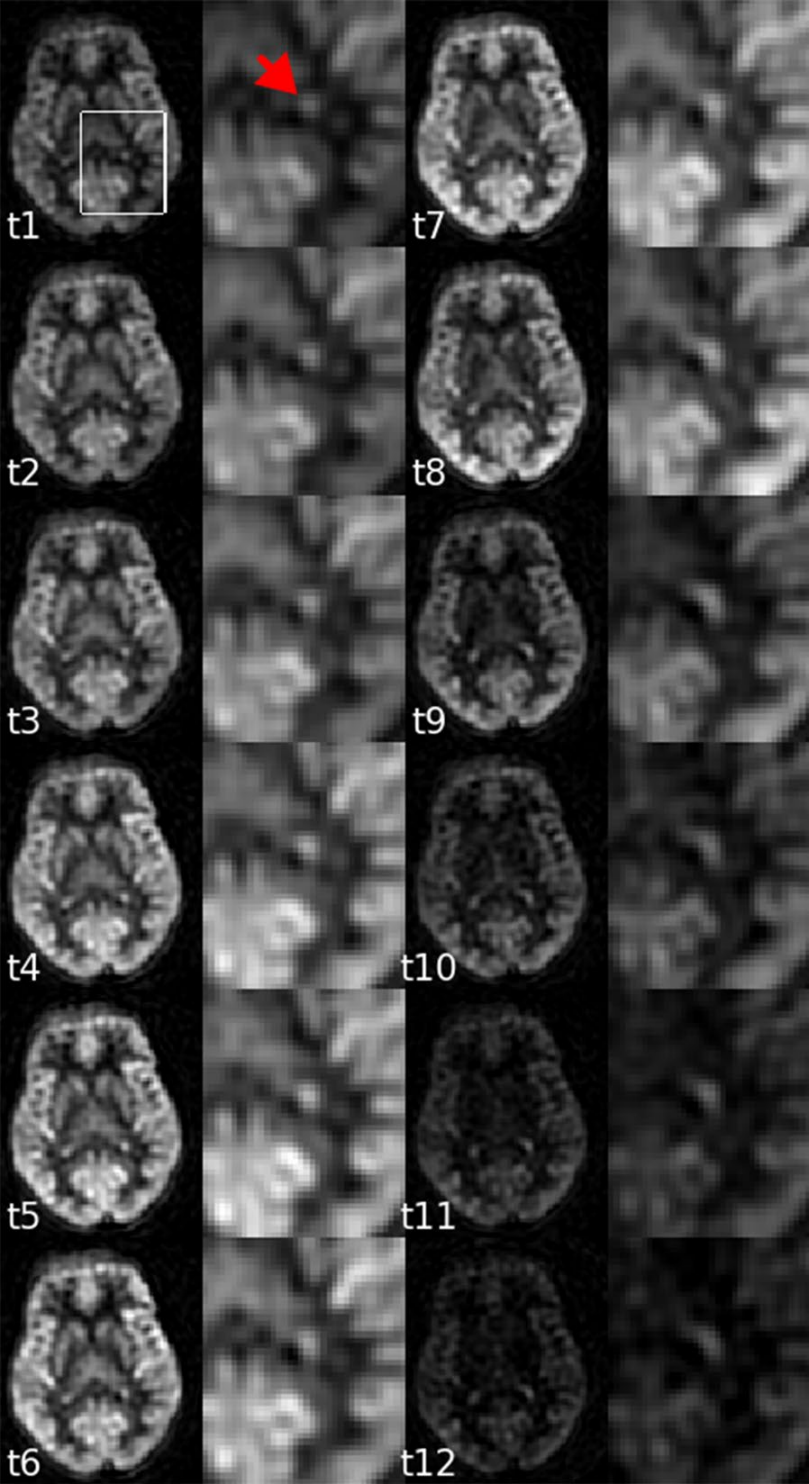
Relative perfusion images at multiple OTs. The choroid plexus regions become more prominent comparing to the gray matter in the scans with the longer blood labeling and longer post labeling delay. The choroid plexus region is highlighted by the red arrow

ASL signal in the choroid plexus and the brain was readily detected and qualitatively consistent with expected behavior. The relative perfusion images, $\Delta M/M_{0}$, show the perfusion evolution across multiple OTs, Fig. 2. In the first six OTs, when longer blood labeling duration was used, the ASL signal of the gray matter increased. This was because more labeled blood flowed into the brain tissue and had stronger effects than the ASL signal decay. In the later six OTs, when the PLD further increased, the ASL signal reduced when the labeled blood signal recovered towards the control blood value.

The red arrow in Fig. 2 highlights the prominent signal of the choroid plexus. In the early perfusion phases, the signal of choroid plexus was readily noticeable, but did not stand out compared to the gray matter and the thalamus. In the late perfusion phases, the ASL signal of the choroid plexus became more obvious. This indicates that the ASL signal of the choroid plexus had a slower signal decay compared to surrounding tissue. Our results at t8 and t9 showed a similar contrast between the choroid plexus and other regions as previous ASL studies with similar post-labeling delays [26–28].

**Fig. 3:**
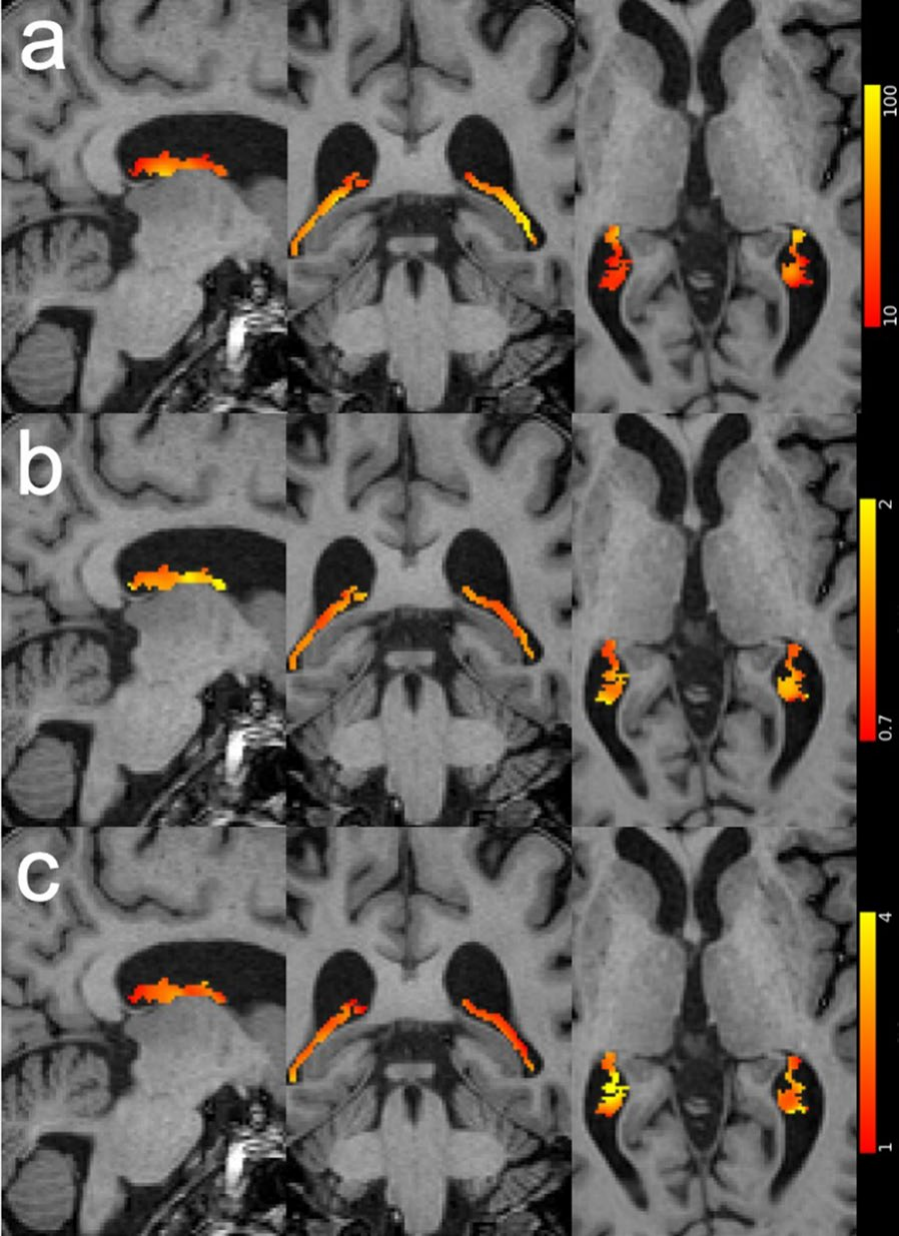
Spatial distributions of the choroid plexus ASL measures overlaid on the T1 weighted images of a selected volunteer. **a** apparent blood flow (ml/100 g/min), **b** arterial transit time (s), and **c** longitudinal relaxation time T_1_ (s)

Figure 3 shows the spatial distributions of the choroid plexus perfusion characteristics on the same subject and the same location as Fig. 1. The junction between the choroid plexus and the lateral ventricle walls showed higher apparent blood flow, compared to other parts, as shown in Fig. 3 the right column. It may be caused by partial volume effects. The junction also showed a lower arterial transit time, which may be consistent with the arterial blood transit from the ventricle walls. The spatial parameter distributions of the other subjects were provided in Additional file 1: Figures.

**Fig. 4:**
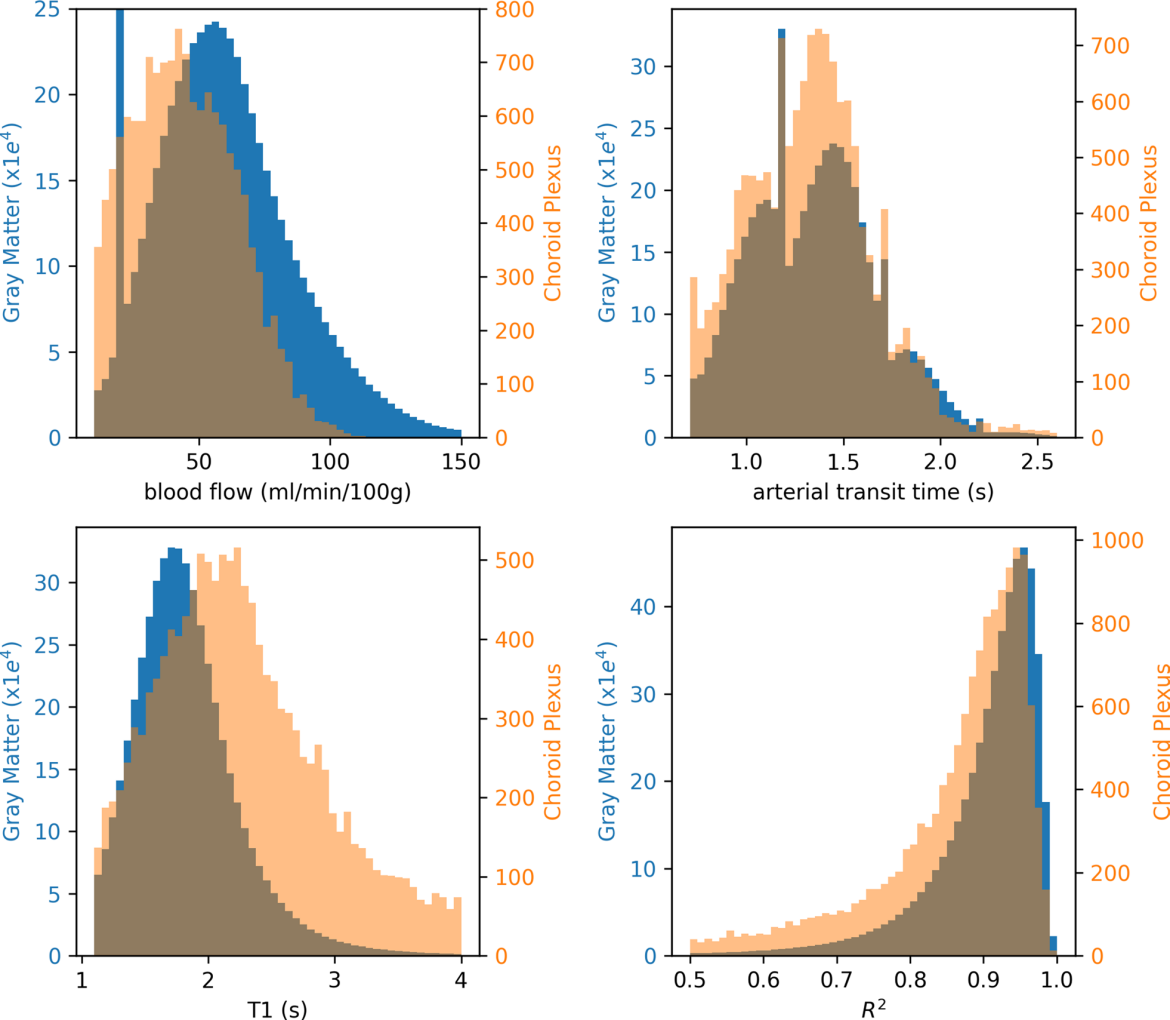
Histograms of voxel-wise ASL measures in choroid plexus and gray matter of 7 volunteers

The voxel-wised distributions of the apparent blood flow, arterial transit time, T_1_ values, and coefficients of determination $R^{2}$ from all 7 subjects are shown in Fig. 4. The apparent blood flow and the T_1_ showed distinguishable distributions between the gray matter and the choroid plexus, although voxel-vise perfusion contained higher noise than the average regional perfusion below.

In the average perfusion characteristics, the choroid plexus regions showed significantly lower apparent blood flow (p < 0.02) and significantly longer T_1_ (p < 0.02) than the gray matter across subjects, Table 2. Across the subjects, the volume of choroid plexus in the lateral ventricles was 2.08 ± 0.56 ml, which was consistent with previous studies using MRI [22, 48], and the total blood flow was 0.80 ± 0.31 ml/min. However, high-resolution T_1_ weighted MRI also contains partial volume effects. The choroid plexus was reported to be about 2 g, and about 50% of the total choroid plexus is located in the lateral ventricles [2]. By correcting these factors, the blood flow of the choroid plexus can be 80 ml/100 g/min, yielding about 1.6 × blood flow of the gray matter.

**Table 2:** Perfusion characteristics of the choroid plexus across subjects

Subject	Gray matter			Choroid plexus		
	Apparent blood flow (ml/100 g/min)	Transit time (s)	T_1_ (s)	Apparent blood flow (ml/100 g/min)	Transit time (s)	T_1_ (s)
1	42.55	1.37	1.93	39.73	1.35	2.45
2	63.70	1.16	1.83	27.40	0.83	2.85
3	61.30	1.12	1.83	58.13	1.27	1.94
4	56.33	1.29	1.77	46.03	1.10	2.32
5	54.65	1.35	1.71	38.47	1.43	1.90
6	47.04	1.45	2.02	40.24	1.45	2.42
7	50.63	1.41	1.85	26.40	1.23	2.42
Mean	53.74 ± 7.01*	1.31 ± 0.12	1.85 ± 0.10*	39.48 ± 10.07*	1.24 ± 0.20	2.33 ± 0.30*

^*^p < 0.02, significant difference between the gray matter and the choroid plexus

**Fig. 5:**
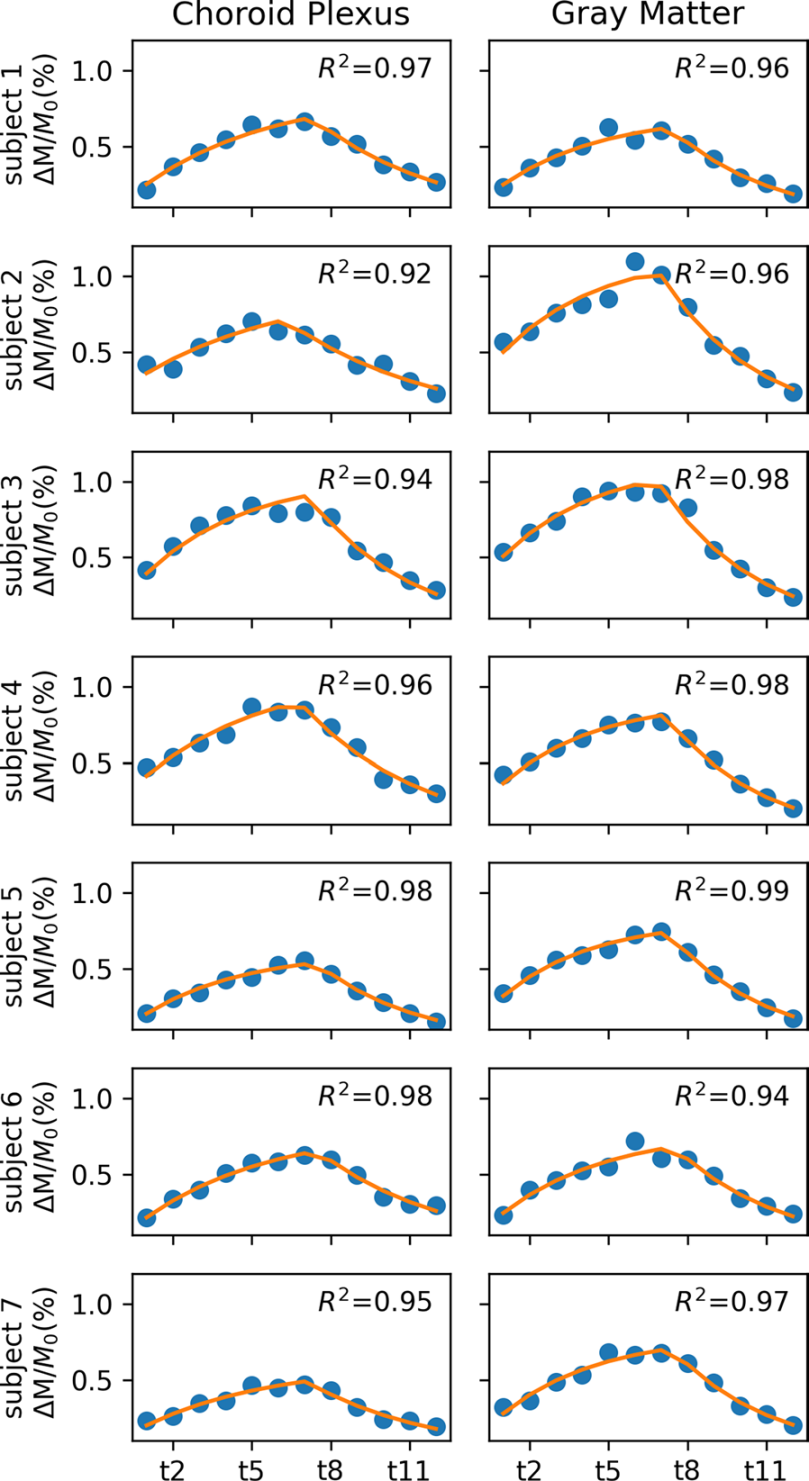
Perfusion model fitting in gray matter and choroid plexus regions

High coefficients of determination ($R^{2}$) were shown in the perfusion model fitting, Fig. 5. Across the subjects, the R^2^ of gray matter regions was 96.89 ± 1.47% and that of the choroid plexus regions was 95.65 ± 2.20%. It indicated high accuracy in perfusion quantification and no significant difference (p = 0.24) of the accuracy between the two regions.

## Discussion

In this work, we quantified the dynamic characteristics of the choroid plexus perfusion using ASL. This work demonstrated the value of ASL as a noninvasive tool to evaluate the choroid plexus perfusion. The apparent blood flow and T_1_ in the choroid plexus are significantly different from the gray matter.

This work provided the first non-invasive measures of the choroid plexus perfusion characteristics. Average apparent blood flow of 39.5 ml/100 g/min and total blood flow of 0.8 ml/min was found at choroid plexus in the lateral ventricle. While choroid plexus perfusion was characterized using microsphere tracers and ex-vivo animal models in the early studies [15–17], they were not able to provide spatial distributions of blood flow or dynamic perfusion information, such as arterial transit time. Our study clearly demonstrated the kinetics of perfusion labeled signal and its spatial distribution. The apparent blood flow measured in this work was in agreement with Johnson et al.’s ASL study [33].

The choroid plexus blood flow of this work should be interpreted as apparent perfusion because of the partial volume effect in ASL images. Recent work showed consistent results between ASL and radiograph on rat brains [49], and our approach in this work was consistent with previous human studies [33, 35]. Therefore, equivalent detectability was expected in this ASL study. However, our work showed relatively lower blood flow in the choroid plexus to that of the cerebrum, compared to previous animal models. The major reason is partial volume effects. Therefore, we reported the total blood flow and adapted a correction factor based on the choroid plexus weight and percentile in the lateral ventricles, yielding that choroid plexus blood flow was 1.6 times higher than the gray matter. Pixel-wise partial volume measurement will provide a more accurate calibration of the blood flow at the choroid plexus regions, which is worth further investigation. A further possible reason is the variations among species. The ratio between choroid plexus blood flow and cerebrum blood flow was about three [19, 21] or five [20] in rats, four in monkey, seven in dogs [16], and about ten in rabbits [17] and dogs [18]. Although detailed studies have been performed on animal models under anesthesia, the availability of human choroid plexus data (e.g., weight and blood flow) is very limited.

Total blood flow represents the function of the choroid plexus more accurately than apparent blood flow maps. Because of the low resolution of ASL images and the sheet-like structure of the choroid plexus, ASL images have considerable partial volume effects, which resulted in a reduced ASL signal at each voxel. In addition, assuming that a fraction of labeled blood water was transferred into CSF, the surrounding CSF generated from the labeled blood can also contribute to the ASL signal. The existing CSF without water transfer has no ASL signal and resulted in the partial volume effect. Since the volume percentile of the choroid plexus and generated CSF is unknown and can be different at each voxel, the measurement at the voxel level is an apparent blood flow, which is not accurate. However, the total ASL signal across the choroid plexus region is not influenced by partial volume effects, so the total blood flow provides the choroid plexus blood flow more accurately. Total blood flow of the choroid plexus showed about 38% variance, which could be from the inter-subject variation, including the choroid plexus volume variation and the average blood flow variation. Our results showed about 25% variation for either, which was consistent with the previous studies [22, 33].

Although partial volume effects reduced the signal-to-noise ratio and the accuracy of perfusion quantification at the choroid plexus, there was no significant accuracy difference from the gray matter in this study. Early ASL studies showed overestimated blood flow and reduced accuracy at low blood flow comparing to the quantitative radiography [50, 51] and PET measurements [52]. This problem can be caused by the underestimated arterial transit time. The latest developments of pCASL have shown the ability to detect the blood flow as low as 15 ml/100 g/min [53] and consistent quantification as in quantitative radiography [49]. The ASL used in this study had similar features as the above two latest developments. Therefore, a similar accuracy was expected in this work. The dynamic signal curve in Fig. 5 further demonstrates the ability of ASL to detect the blood flow at the choroid plexus. The coefficients of determination in model fitting showed high accuracy and no significant difference between the gray matter and the choroid plexus regions.

Another potential cause of underestimation error in the measurement of blood flow is a lower T_2_* and T_2_ in the choroid plexus than in the CSF and brain tissue. A large fraction of the choroid plexus water volume is blood and this blood likely rapidly equilibrates through the CSF to near the venous oxygenation level [54]. At venous oxygenation levels, the signal in blood decays rapidly with echo time and even refocusing pulses are ineffective at preserving the signals unless very short spacings are used [55]. Future studies using shorter refocusing echo times may be merited to more accurately map and quantify choroid plexus blood flow.

The longer T_1_ at the choroid plexus may be evocative of CSF generation. A slow ASL signal decay was noted at the choroid plexus compared to the gray matter. Table 2 shows that the T_1_ of the choroid plexus was longer than the gray matter and the arterial blood. Since CSF is the only brain tissue with a T_1_ longer than 2 s, this result may suggest that the labeled water transfers from arterial blood to CSF. Therefore, ASL may provide a feasible method to detect CSF generation, but further investigation is required. For example, recent work showed that increases of choroid plexus weight coexisted with decreases of CSF generation [56]. This may suggest a complex relationship between choroid plexus perfusion, mass, and CSF generation.

Several limitations of this study should be noted. First, the cohort size of this work was relatively small. The variances of the blood flow and the choroid plexus volume result in a large variation in blood flow estimation. More reliable reference characteristics of the choroid plexus perfusion can be obtained with increased sample size and age distribution of controls. Second, this study focused on the choroid plexus in the lateral ventricles. The choroid plexus in the 3rd and 4th ventricles will require further investigation. Third, the choroid plexus region selection on the ASL images has limited accuracy. The choroid plexus region was transferred from T_1_ weighted images to the ASL domain based on the volume registration between ASL and the gray matter. However, this may not be an effective registration for the choroid plexus region, and the registration quality can be limited by the ASL resolution. A surface constrained registration [57] and high-resolution ASL images [58] may provide improved accuracy. Fourth, current work cannot exclude the impact of the different feeding arteries of the gray matter and the choroid plexus. Arterial transit time was reported to be shorter in the deep gray matter with ASL [59]. Therefore, further evaluations are required to understand the transit time at the choroid plexus. Finally, a number of parameters (e.g., relaxation times) were approximated or assumed in the quantification. Since these parameters were consistent between the gray matter and choroid plexus perfusion quantifications, similar effects were expected. Furthermore, these parameters were global scale factors in the ASL mode, which will influence the estimated apparent blood flow value on a similar level as other ASL studies, but have negligible effects on the T_1_ and arterial transit time quantification. Therefore, we anticipate that the errors from these approximations will not affect the T_1_ differences between the gray matter and choroid plexus regions. Further studies aimed at detailed quantification models for choroid plexus will be merited if the measure proves useful.

## Conclusion

This work demonstrated arterial spin labeling MRI as a feasible and safe tool for choroid plexus perfusion studies. The dynamic ASL method is readily feasible to use and can help studies of choroid plexus lesions and function. This work also provided useful reference values of the choroid plexus perfusion characteristics. Compared to the gray matter, the choroid plexus has significantly longer T_1_, which may indicate transport of the label into CSF by diffusion or CSF generation.

## Supplementary information

**Additional file 1: Figures.** Spatial distributions of the choroid plexus ASL measures overlaid on the T_1_ weighted images of the other volunteers. (a) apparent blood flow (ml/100g/min), (b) arterial transit time (s) and (c) longitudinal relaxation time T_1_ (s).

## Data Availability

The datasets generated and/or analyzed during the current study are not publicly available due to IRB regulation, but are available from the corresponding author on reasonable request.

## Abbreviations

CSF: Cerebrospinal fluid
AD: Alzheimer’s Disease
MRI: Magnetic resonance imaging
ASL: Arterial spin labeling
pCASL: Pseudo-continuous arterial spin labeling
LD: Labeling duration
PLD: Post-labeling delay
OT: Observation time
