# Supplementary figures and images for: Non-invasive measurement of choroid plexus apparent blood flow with arterial spin labeling
Source: Fluids Barriers CNS. 2020 Sep 22;17:58. doi: 10.1186/s12987-020-00218-z (PMC7510126; doi:10.1186/s12987-020-00218-z)

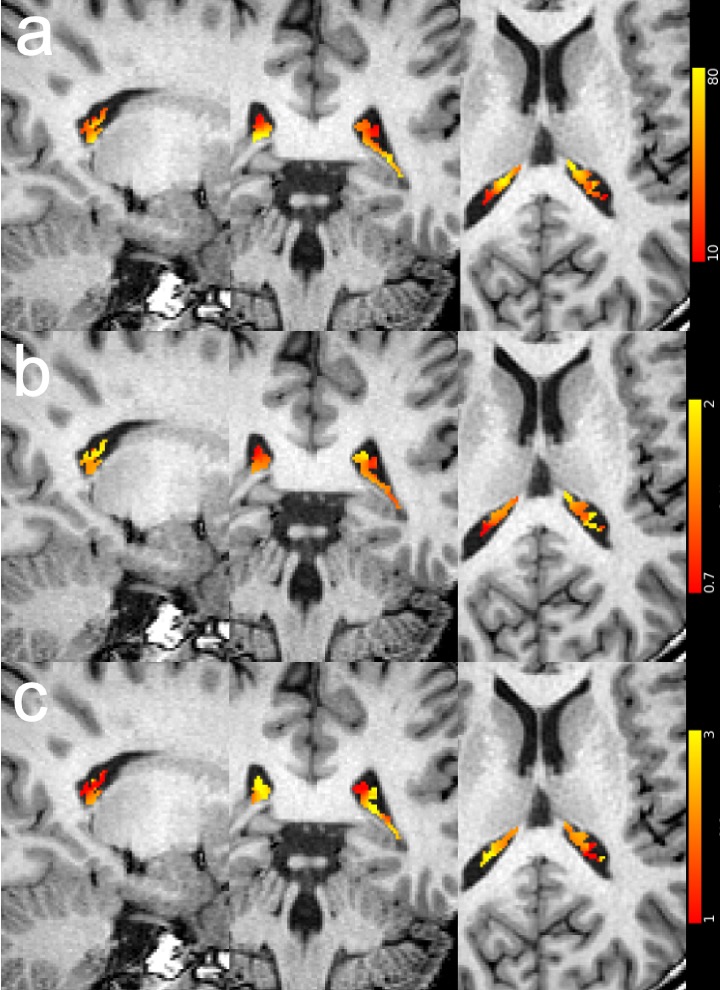

Supplement: Supplementary file 1 — Additional file 1: Figures. Spatial distributions of the choroid plexus ASL measures overlaid on the T1 weighted images of the other volunteers. (a) apparent blood flow (ml/100g/min), (b) arterial transit time (s) and (c) longitudinal relaxation time T1 (s). [file 12987_2020_218_MOESM1_ESM.zip › 12987_2020_218_MOESM1_ESM.jpg]

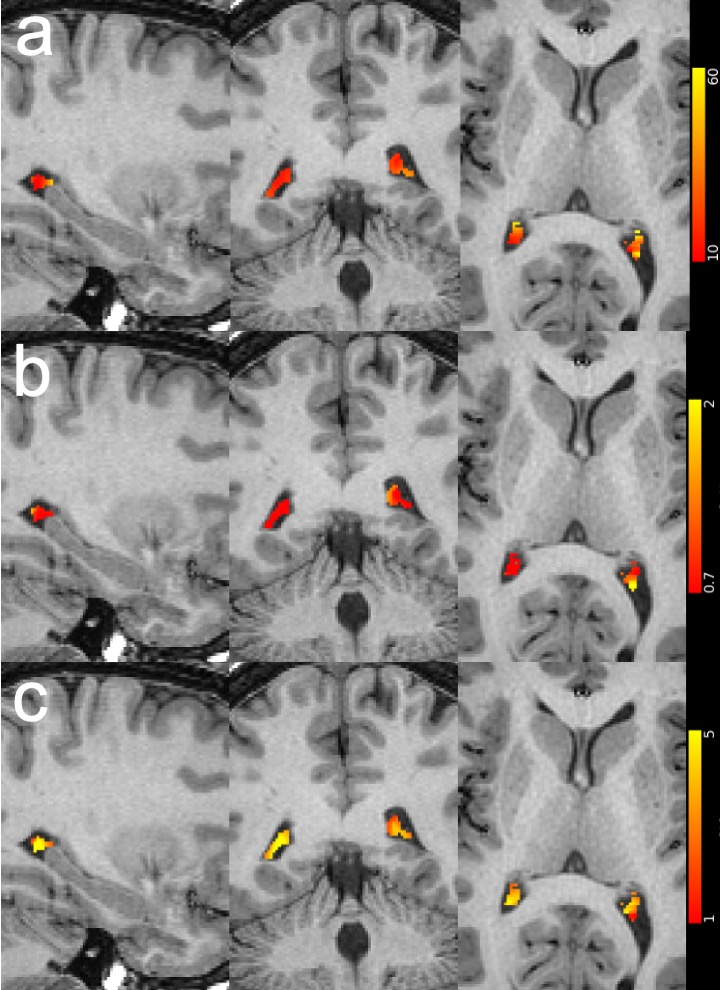

Supplement: Supplementary file 1 — Additional file 1: Figures. Spatial distributions of the choroid plexus ASL measures overlaid on the T1 weighted images of the other volunteers. (a) apparent blood flow (ml/100g/min), (b) arterial transit time (s) and (c) longitudinal relaxation time T1 (s). [file 12987_2020_218_MOESM1_ESM.zip › 12987_2020_218_MOESM2_ESM.jpg]

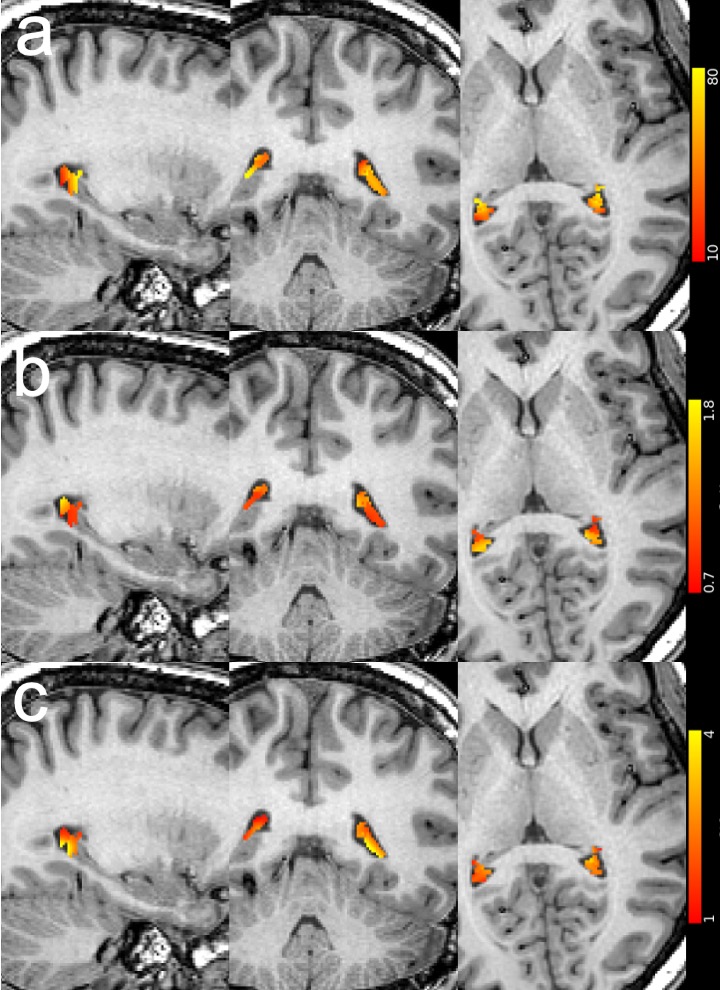

Supplement: Supplementary file 1 — Additional file 1: Figures. Spatial distributions of the choroid plexus ASL measures overlaid on the T1 weighted images of the other volunteers. (a) apparent blood flow (ml/100g/min), (b) arterial transit time (s) and (c) longitudinal relaxation time T1 (s). [file 12987_2020_218_MOESM1_ESM.zip › 12987_2020_218_MOESM3_ESM.jpg]

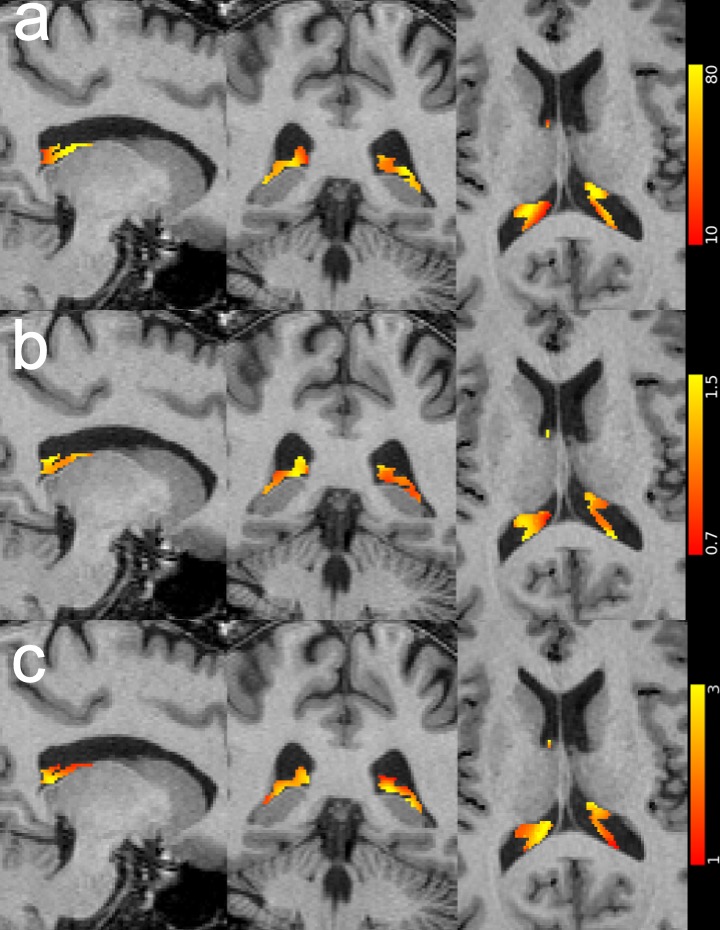

Supplement: Supplementary file 1 — Additional file 1: Figures. Spatial distributions of the choroid plexus ASL measures overlaid on the T1 weighted images of the other volunteers. (a) apparent blood flow (ml/100g/min), (b) arterial transit time (s) and (c) longitudinal relaxation time T1 (s). [file 12987_2020_218_MOESM1_ESM.zip › 12987_2020_218_MOESM4_ESM.jpg]

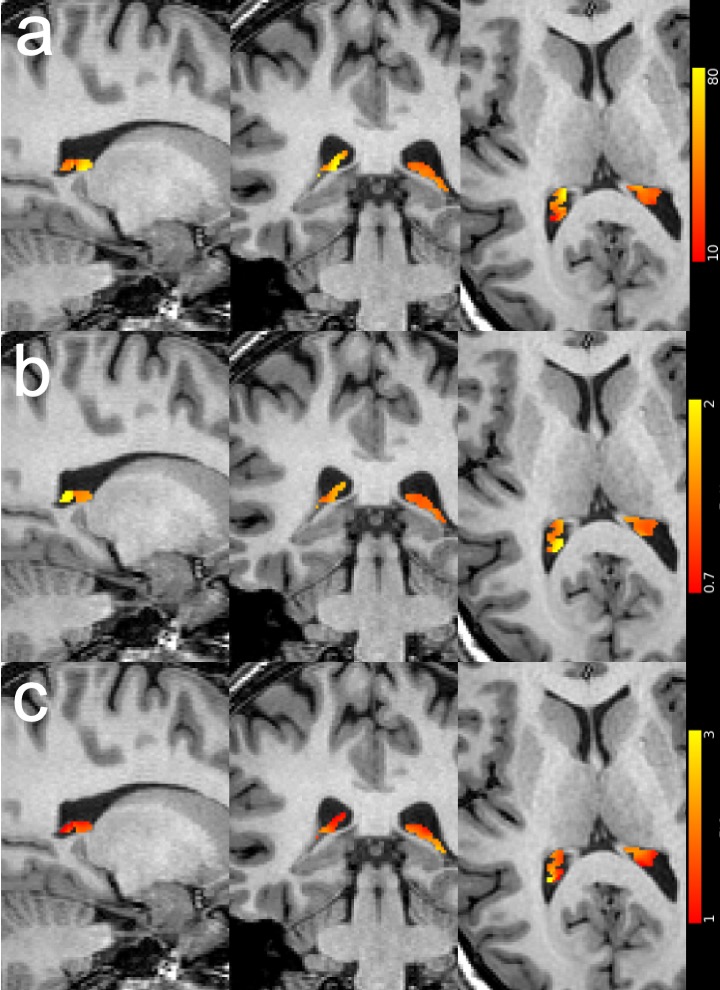

Supplement: Supplementary file 1 — Additional file 1: Figures. Spatial distributions of the choroid plexus ASL measures overlaid on the T1 weighted images of the other volunteers. (a) apparent blood flow (ml/100g/min), (b) arterial transit time (s) and (c) longitudinal relaxation time T1 (s). [file 12987_2020_218_MOESM1_ESM.zip › 12987_2020_218_MOESM5_ESM.jpg]

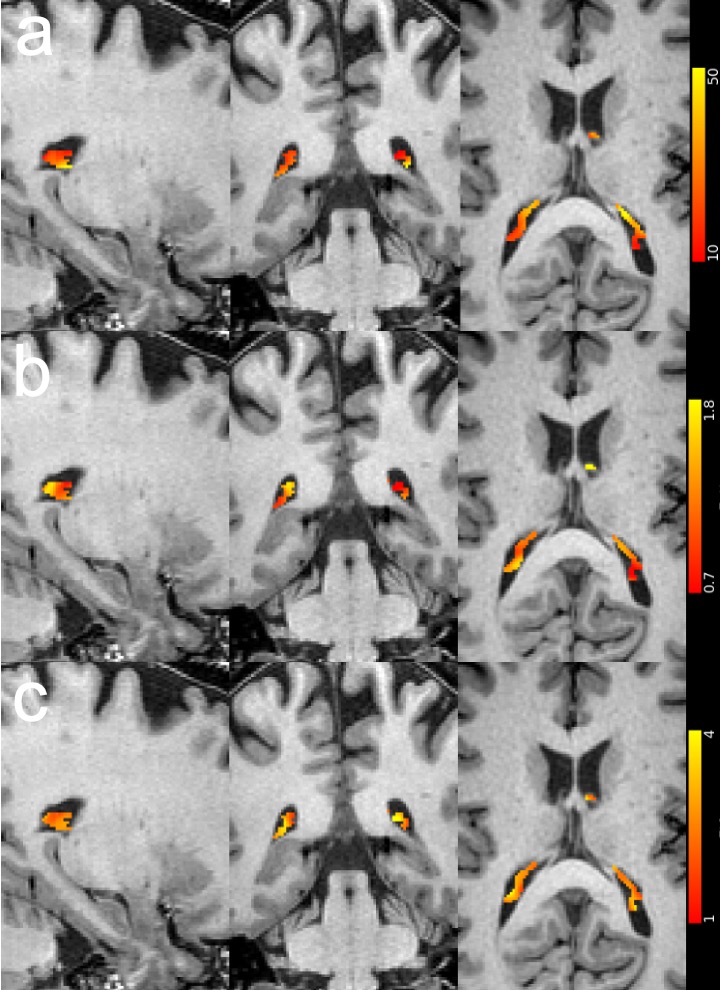

Supplement: Supplementary file 1 — Additional file 1: Figures. Spatial distributions of the choroid plexus ASL measures overlaid on the T1 weighted images of the other volunteers. (a) apparent blood flow (ml/100g/min), (b) arterial transit time (s) and (c) longitudinal relaxation time T1 (s). [file 12987_2020_218_MOESM1_ESM.zip › 12987_2020_218_MOESM6_ESM.jpg]
